# RIF1 controls replication initiation and homologous recombination repair in a radiation dose-dependent manner

**DOI:** 10.1242/jcs.240036

**Published:** 2020-06-22

**Authors:** Yuichiro Saito, Junya Kobayashi, Masato T. Kanemaki, Kenshi Komatsu

**Affiliations:** 1 Department of Genome Repair Dynamics, Radiation Biology Center, Kyoto University, Yoshida Konoe, Sakyo-ku, Kyoto 606-8501, Japan; 2 Department of Chromosome Science, National Institute of Genetics, Mishima, Shizuoka 411-8540, Japan; 3 Department of Genetics, SOKENDAI, Mishima, Shizuoka 411-8540, Japan

**Keywords:** DNA repair, Homologous recombination, Radiation biology, RIF1

## Abstract

RIF1 controls both DNA replication timing and the DNA double-strand break (DSB) repair pathway to maintain genome integrity. However, it remains unclear how RIF1 links these two processes following exposure to ionizing radiation (IR). Here, we show that inhibition of homologous recombination repair (HRR) by RIF1 occurs in a dose-dependent manner and is controlled via DNA replication. RIF1 inhibits both DNA end resection and RAD51 accumulation after exposure to high doses of IR. Contrastingly, HRR inhibition by RIF1 is antagonized by BRCA1 after a low-dose IR exposure. At high IR doses, RIF1 suppresses replication initiation by dephosphorylating MCM helicase. Notably, the dephosphorylation of MCM helicase inhibits both DNA end resection and HRR, even without RIF1. Thus, our data show the importance of active DNA replication for HRR and suggest a common suppression mechanism for DNA replication and HRR at high IR doses, both of which are controlled by RIF1.

This article has an associated First Person interview with the first author of the paper.

## Abstract (precis)

**Summary:** Homologous recombination repair (HRR) is suppressed by radiation exposure in a dose-dependent manner. RIF1 has a role in this dose-dependent suppression of HRR by controlling replication initiation.

## INTRODUCTION

The exposure of cells to ionizing radiation (IR) induces DNA double-strand breaks (DSBs), which are severely deleterious lesions that may induce reproductive cell death and lead to a predisposition to tumors (Löbrich and Jeggo, 2007). DSBs are largely rejoined through two major repair pathways, non-homologous end-joining (NHEJ) or homologous recombination repair (HRR). HRR is initiated with 5′ DNA end resection at DSB sites and uses undamaged homologous regions, generally from the sister chromatid, as template for the action of RAD51. The current view of the decision-making process that determines choice of DSB repair pathway is that it depends on the cell cycle, because HRR was suggested to be active only in the S and G2 phases, during which the sister chromatids are available, whereas NHEJ could be activated throughout the cell cycle. This cell cycle dependence of HRR was evidenced by the fact that CDK promotes HRR by phosphorylating HRR proteins (Esashi et al., 2005; Ira et al., 2004).

Previous studies have shown that the choice between NHEJ and HRR repair pathways is regulated by the 53BP1 (also known as TP53BP1)–BRCA1 circuit (Chapman et al., 2013; Di Virgilio et al., 2013; Escribano-Díaz et al., 2013; Zimmermann et al., 2013). RIF1 (Rap1-interacting factor 1), the effector of 53BP1, accumulates at DSB sites via the ATM-dependent phosphorylation of 53BP1 and inhibits HRR by protecting DSB ends from DNA end resection. As a result, RIF1 plays a role in directing the cell towards the use of NHEJ, which is the dominant pathway in G1 phase. On the other hand, BRCA1 inhibits RIF1 accumulation during S and G2 phases, resulting in HRR activation. These studies provided a molecular mechanism for the cell-cycle-dependent choice of repair pathway. However, a recent study showed that the phosphorylation of 53BP1 and the resulting formation of RIF1 foci were observed at early time points after 2 Gy of IR even in G2-phase cells, indicating a previously unrevealed mechanism of HRR regulation by the 53BP1–BRCA1 circuit (Isono et al., 2017).

In addition to acting in the repair pathway choice, RIF1 plays a major role in the regulation of replication timing during S phase, which appears to be a conserved function for RIF1 throughout eukaryotes (Hayano et al., 2012; Ira and Nussenzweig, 2014). Replication initiation begins at replication origins, where the MCM (minichromosome maintenance) helicase complex, composed of MCM2–7, is formed through the stepwise assembly of the origin recognition complex (ORC), CDC6, CDT1, and MCM during the G1 phase. Once S phase is initiated, the MCM helicase is converted into its active form in the CDC45–MCM–GINS complex (CMG complex), for which MCM2 and MCM4 are phosphorylated by Dbf4-dependent kinase (DDK) composed of CDC7 and DBF4 (Chuang et al., 2009; Kang et al., 2012). Simultaneously, RIF1 forms a complex with protein phosphatase 1 (PP1), which has poor substrate specificity and requires association with a targeting subunit for the correct substrate recognition. The RIF1–PP1 module acts as a suppressor of replication initiation by dephosphorylating MCM2 and MCM4, which maintains MCM helicase in an inactive state until cells are ready to initiate replication at each origin during S phase (Alver et al., 2017; Hiraga et al., 2014; Hiraga et al., 2017; Yamazaki et al., 2012).

There are intimate links between HRR and replication, in which HRR is critical for the successful completion of replication. In bacteria, recombination-directed D-loop formation can initiate DNA replication in the absence of origins (Kogoma, 1997). This mechanism seems to be conserved in eukaryotes as break-induced replication (BIR) for collapsed DNA replication forks (Anand et al., 2013). BIR is well characterized in budding yeast as a non-canonical HRR pathway, specifically for one-ended DSBs. In mammalian cells, RAD52 is recruited to sites of replication stress and is required for fork restart by BIR (Sotiriou et al., 2016). Although these studies showed a pivotal role for HRR in restarting replication at one-ended DSBs, the relationship between HRR and replication after IR exposure, which induces two-ended DSBs, is largely unknown.

In order to understand the regulatory mechanisms of HRR following IR exposure, we quantified HRR activity after exposure to IR and demonstrated an IR dose-dependent suppression of HRR, mediated by RIF1. RIF1 inhibited the accumulation of RAD51 at DSB sites after 3 Gy of IR but not after 0.5 Gy. DR-GFP, an HRR reporter gene, showed that RIF1 inhibited HRR only after high IR doses. In these cases, RIF1 accumulated at DSB sites and led to a temporary suppression of replication by promoting dephosphorylation of MCM helicase. Interestingly, dephosphorylation of MCM helicase by a DDK inhibitor or expression of a phospho-dead MCM2 mutant suppressed RAD51 accumulation and HRR at DSB sites. Our results suggest that efficient HRR following IR exposure requires active replication, while RIF1 suppresses replication and HRR in an IR dose-dependent manner.

## RESULTS

### HRR is inhibited by IR in a dose-dependent manner, whereas NHEJ remains unchanged

**Fig. 1.**
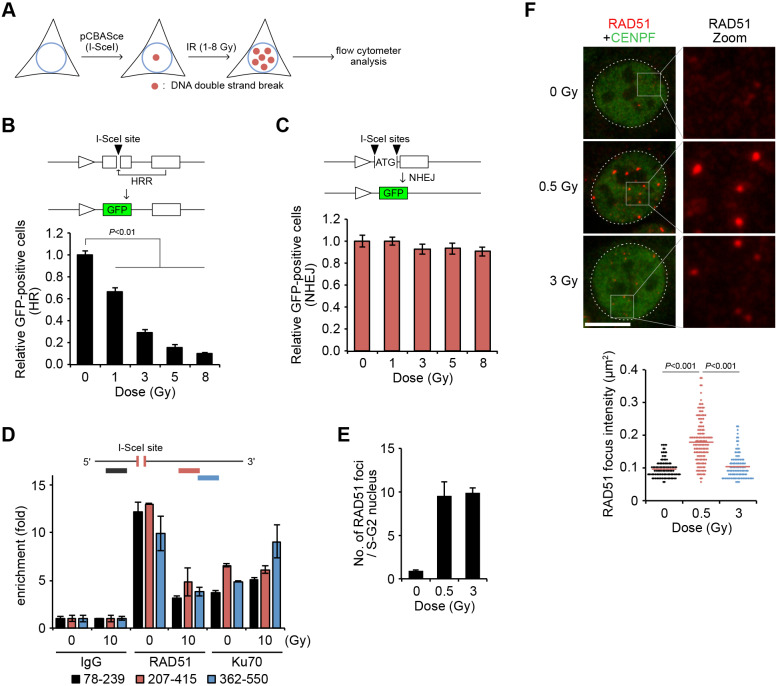
**Radiation inhibits HRR in a dose-dependent manner.** (A) Schematic illustration of the HRR (DR-GFP) and NHEJ (pEJ) assays after irradiation (IR). GFP-positive cells were scored as HRR or NHEJ frequencies. (B) Decrease in HRR after irradiation. HeLa cells were transfected with pCBASce and irradiated with the represented dose of IR 4 h after the transfection. The frequency of GFP-positive cells was normalized to an un-irradiated control. Data represent the mean±s.d. of three independent experiments. (C) No variations were seen in the NHEJ frequency after irradiation. HeLa cells carrying the pEJ reporter gene were prepared and analyzed as described for B. Data represent the mean±s.d. of three independent experiments. (D) Decrease in RAD51 accumulation but not in Ku70 accumulation. ChIP assays using antibodies against RAD51, Ku70 and IgG control were performed with extracts from U2OS cells, which were transfected with pCBASce and then irradiated with 10 Gy. The enrichment of DNA was determined by qPCR using primers that annealed at the indicated nucleotide distances from an I-SceI site. Data represent the mean±s.d. of two independent experiments. (E) Number of RAD51 foci per nucleus after irradiation. The irradiated cells were fixed 1 h after irradiation and were stained with anti-RAD51 antibodies. Data represent the mean±s.d. of three independent experiments. (F) Signal intensity of individual RAD51 foci (red) measured in E. Nuclei are stained with anti-CENPF (green) and nuclear boundaries are marked with dotted lines. Boxes indicate regions shown in magnified views. Each dot in the bottom panel represents a single RAD51 focus and horizontal bars indicate the mean intensity. Indicated *P* values were calculated using the Mann–Whitney *U*-test). Scale bar: 10 μm.

To functionally assess the extent of HRR and NHEJ activities in the presence of DSBs, HeLa cells harboring a single copy of either DR-GFP (an HRR reporter gene) or pEJ (an NHEJ reporter gene) were transiently transfected with an I-SceI expression vector and exposed to IR (Fig. 1A). I-SceI induced a single two-ended DSB in each specific reporter gene, and the number of GFP-positive cells was used as a measure of functional HRR or NHEJ repair (Mansour et al., 2008; Pierce et al., 2001). The DR-GFP assay showed a reduction in HRR, whose frequencies were reduced to 30% and 10% of that of the non-irradiated control after IR exposures of 3 and 8 Gy, respectively (Fig. 1B). IR did not affect transfection efficiency or gene expression (Fig. S1A), but significantly inhibited HRR in a dose-dependent manner. In contrast, the amount of NHEJ repair was almost constant, even after 8 Gy of IR (Fig. 1C). Similar results were obtained with human U2OS cells (Fig. S1B,C). This decrease in functional HRR was confirmed using a chromatin immunoprecipitation (ChIP) assay at an I-SceI DSB site (Niida et al., 2010). The accumulation of RAD51, a recombinase involved in sister chromatid exchanges, was strongly suppressed after IR exposure (Fig. 1D). Contrastingly, the accumulation of Ku70 (XRCC6), an NHEJ protein, remained relatively constant. Thus, the functional HRR and ChIP assays for a two-ended DSB at a specific locus showed a decrease in HRR activity with increasing IR doses.

Next, RAD51 focus formation and the activation of DNA-PKcs (also known as PRKDC), a kinase involved in NHEJ, were measured in the absence of reporter genes. Phosphorylation of DNA-PKcs increased along with the IR doses (Fig. S1D). In contrast, the number of RAD51 foci per cell was constant regardless of the IR dose (either 0.5 or 3 Gy), even though 3 Gy of IR produced more DSBs than 0.5 Gy (Fig. 1E). Interestingly, the signal intensity of individual RAD51 foci 1 h after IR exposure was significantly weaker in cells irradiated with 3 Gy of IR than in those irradiated with 0.5 Gy of IR (Fig. 1F; Fig. S1E). This is consistent with a previous finding obtained using quantitative image-based cytometry, which showed a reduction in RAD51 focus intensity after increasing IR doses (Ochs et al., 2016). Our results showed that radiation treatment, which generates additional DSBs, inhibits RAD51-mediated HRR in an IR dose-dependent manner both at an I-SceI-induced DSB and at IR-induced DSBs.

### IR inhibition of HRR is mediated by RIF1

**Fig. 2.**
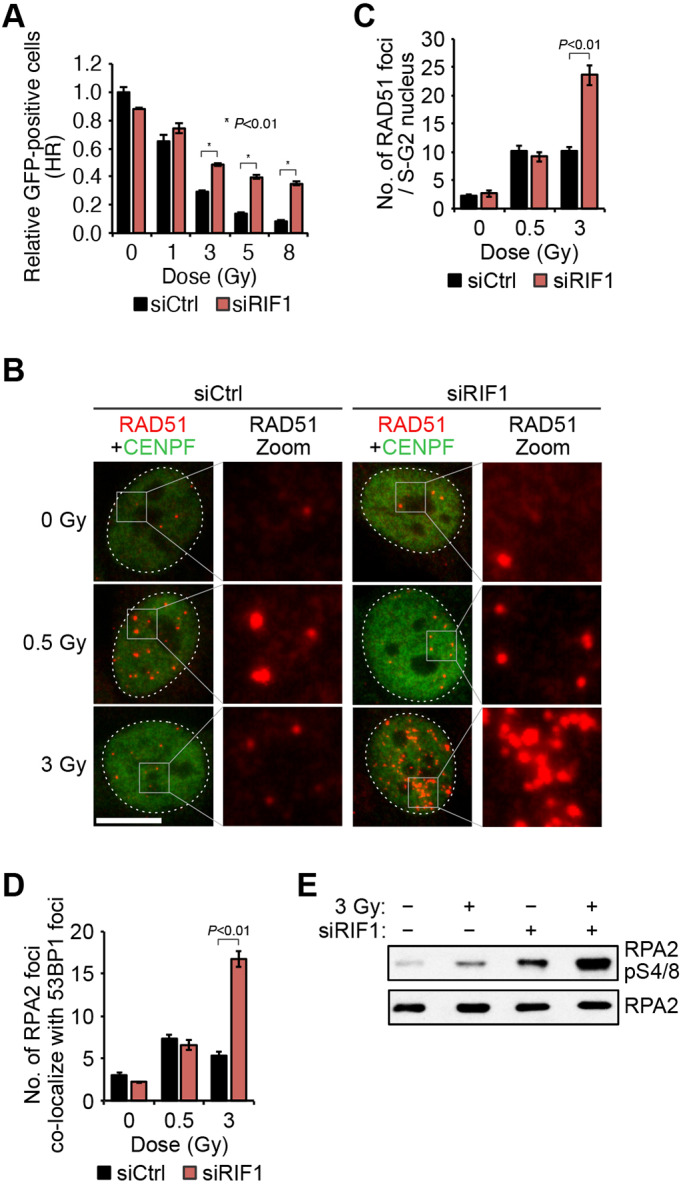
**RIF1 inhibits HRR after exposure to high IR doses but not after low IR doses.** (A) Restoration of radiation-induced HRR inhibition by RIF1 depletion. HeLa cells transfected with siCtrl or siRIF1 were analyzed with the DR-GFP assay as in Fig. 1B. (B,C) Restoration of 3 Gy-induced RAD51 foci by RIF1 depletion. HeLa cells transfected with siCtrl or siRIF1 were stained for RAD51 focus formation 1 h after exposure to 0.5 Gy or 3 Gy and the number of RAD51 foci per nucleus quantified. CENPF was used as a marker of the S/G2 cell cycle phases. Nuclear boundaries are marked with dotted lines and boxes indicate regions shown in magnified views. Scale bar: 10 μm. (D) RPA2 focus formation at DSB sites. HeLa cells were fixed 1 h after irradiation and were stained with anti-RPA2 antibodies. 53BP1 foci were used as markers of DSB sites. (E) Increased phosphorylation of RPA2 at Ser4 or Ser8 (pS4/S8) after RIF1 depletion. HeLa cells transfected with siCtrl or siRIF1 were irradiated with 3 Gy and collected 1 h after irradiation, for sample preparation. RPA2pS4/S8 and RPA2 proteins were detected by western blotting. Data in A,C and D are mean±s.d. Indicated *P* values were calculated using a two-way ANOVA, followed by Tukey's multiple comparisons test.

In order to gain insight into the relationship between HRR and NHEJ upon IR treatment, we monitored HRR while inhibiting NHEJ. HeLa-DR-GFP cells were treated with a DNA-PKcs inhibitor after transfection with an I-SceI expression vector and analyzed using a flow cytometer. Unexpectedly, the DNA-PKcs inhibition did not rescue the reduction in HRR after high doses of IR (Fig. S2A). Because RIF1 is proposed to suppress HRR in a different pathway from that of DNA-PKcs, we next measured HRR activity when RIF1 was depleted by siRNA (Fig. S2B). RIF1 depletion alleviated the inhibition of HRR by IR, although this repair pathway was not completely restored after exposure to any radiation dose (Fig. 2A). This partial restoration of functional HRR might be explained by either a hyper-end resection after RIF1 depletion (Fig. 2E, lane 4) (Ochs et al., 2016), or a positive role of RIF1 in HRR, which was observed in non-irradiated cells (Fig. 2A, 0 Gy) (Buonomo et al., 2009). To confirm RIF1 function on HRR suppression, RAD51 focus formation was observed in RIF1-depleted cells. The decrease in RAD51 intensity after an IR exposure of 3 Gy was rescued when RIF1 was depleted (Fig. 2B,C). RIF1 depletion significantly promoted DNA-end resection in cells treated with 3 Gy of IR, which was detected by quantifying formation of foci of RPA2, a single-strand DNA binding protein, and by assaying RPA2 phosphorylation (Fig. 2D,E; Fig. S2C). These results were not caused by an alteration in the cell cycle after RIF1 depletion (Fig. S2D). These findings were consistent with previous studies (Chapman et al., 2013; Di Virgilio et al., 2013; Escribano-Díaz et al., 2013; Zimmermann et al., 2013). However, the enhanced focus formation of RPA2 and RAD51 in RIF1-depleted cells was prominent after exposure to 3 Gy but not 0.5 Gy of IR, suggesting that RIF1 inhibits HRR only at high IR doses.

### BRCA1 antagonizes RIF1 activity after low IR doses during S/G2 phase

**Fig. 3.**
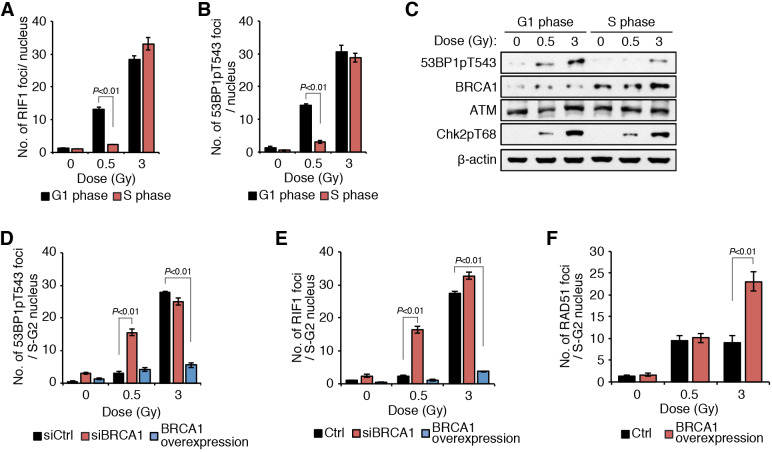
**BRCA1 antagonizes ATM-dependent RIF1 accumulation at low IR doses.** (A,B) Marginal formation of 0.5 Gy-induced foci of RIF1 and phospho-53BP1 in S-phase cells. HeLa cells were synchronized with a double thymidine block in G1 or S phases and were irradiated with 0.5 Gy or 3 Gy of IR. Cells were fixed and stained with anti-RIF1 (A) or anti-phospho-53BP1 (B) antibodies 0.5 h after irradiation and the number of foci per nucleus was quantified. (C) Phosphorylation of 53BP1 and CHK2 after irradiation. HeLa cells synchronized in G1 or S phase were irradiated and collected 1 h after irradiation for western blotting with the indicated antibodies. β-actin is shown as a loading control. (D,E) Phospho-53BP1 (D) or RIF1 (E) foci formation after irradiation in BRCA1-depleted and BRCA1-overexpressing cells. HeLa cells transfected with siCtrl, siBRCA1 or a BRCA1-expressing vector were stained with anti-phospho-53BP1 or anti-RIF1 antibodies 0.5 h after IR exposure of 0.5 Gy or 3 Gy. (F) Enhanced RAD51 focus formation after exposure to 3 Gy with BRCA1 overexpression. HeLa cells transfected with empty or BRCA1-expressing vectors were stained for RAD51 focus formation 1 h after exposure to 0.5 Gy or 3 Gy. Data in A,B,D–F are mean±s.d., and the *P* values indicated were calculated by two-way ANOVA followed by Tukey's multiple comparisons test.

Next, to examine the mechanisms of IR dose-dependent HRR inhibition by RIF1, we analyzed the dose-dependency of 53BP1 phosphorylation, which is a prerequisite for RIF1 accumulation (Chapman et al., 2013; Di Virgilio et al., 2013; Escribano-Díaz et al., 2013; Zimmermann et al., 2013). HeLa cells were synchronized in G1 phase by thymidine block and the arrested cells were released into S phase (Fig. S3A). G1- or S-phase cells were exposed to 0.5 or 3 Gy of IR. In G1-phase cells, phospho-53BP1 and RIF1 formed foci regardless of IR dose (Fig. 3A,B; Fig. S3B,C). By contrast, these foci were negligible after 0.5 Gy, but were evident after 3 Gy of IR in S-phase cells. Importantly, the number of 53BP1 foci formed in both G1 and S phases were similar (Fig. S3D), suggesting that 53BP1 phosphorylation after 0.5 Gy of IR was suppressed in S-phase cells. Because ATM-mediated CHK2 (also known as CHEK2) activation was observed in both G1- and S-phase cells exposed to 0.5 Gy of IR (Fig. 3C), the DNA damage checkpoint is not responsible for the suppression of 53BP1 phosphorylation. These results suggest that, in S phase, 53BP1 phosphorylation is controlled by an unknown mechanism.

BRCA1 was reported to play a suppressive role in RIF1 focus formation by promoting dephosphorylation of phospho-53BP1 (Isono et al., 2017). BRCA1 expression was increased in cells that were synchronized in S phase, in which phospho-53BP1 was suppressed, although the cell cycle synchronization may have artificially affected the BRCA1 expression level (Fig. S3E) (Escribano-Díaz et al., 2013). Therefore, we used siRNA or a BRCA1 overexpression model to test the effect of BRCA1 expression level on phospho-53BP1 and RIF1 accumulation. BRCA1 depletion enhanced the number of phospho-53BP1 and RIF1 foci after 0.5 Gy but not after 3 Gy of IR, suggesting that the endogenous BRCA1 in S-phase cells was sufficient to play an antagonistic role after 0.5 Gy but insufficient after 3 Gy of IR (Fig. 3D,E). This was confirmed by the observation that BRCA1 overexpression reduced the number of phospho-53BP1 and RIF1 foci, leading to an increased number of RAD51 foci after 3 Gy of IR (Fig. 3D–F; Fig. S3F). These results demonstrated that HRR activity was intact after 0.5 Gy of IR due to the prominent antagonistic effect of BRCA1 in S phase, whereas this antagonistic effect was possibly prevented by enhanced activity of checkpoint kinases such as ATM after 3 Gy of IR.

### IR suppresses DNA replication through the accumulation of RIF1

**Fig. 4.**
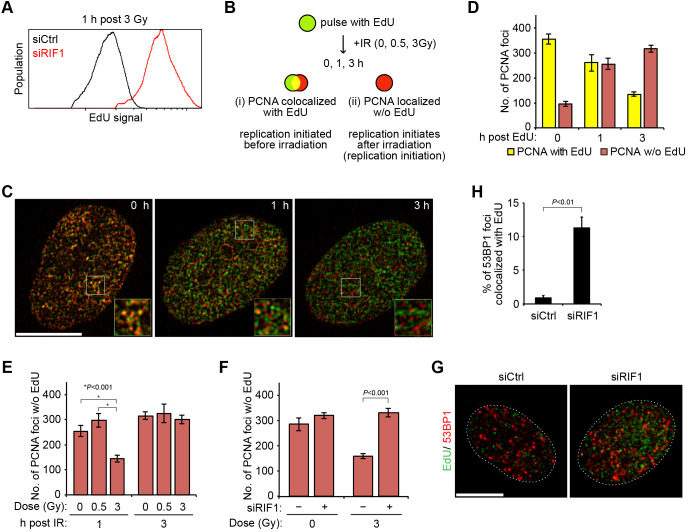
**RIF1 inhibits replication initiation after exposure to high IR doses but not after low IR doses.** (A) DNA synthesis after exposure to 3 Gy IR in siCtrl and siRIF1 cells. Incorporation of EdU was measured 1 h after irradiation using a flow cytometer. (B) Schematic illustration of pulse-labeling and PCNA staining. HeLa cells synchronized in mid-S phase were pulse-labeled with EdU and stained with anti-PCNA antibodies after exposure to 0.5 Gy or 3 Gy of IR. (C,D) Time course of PCNA and EdU foci formation in non-irradiated HeLa cells. PCNA foci, scored as either colocalizing with EdU foci (>0.05 µm^2^ overlap area, PCNA with EdU) or not (<0.05 µm^2^ overlap area, PCNA w/o EdU), were quantified in each nucleus. Data are the mean±s.d. Boxes in C indicate regions shown in magnified images. Scale bar: 10 μm. (E) Transient replication block after exposure to 3 Gy of IR. After pulse-labeling, HeLa cells were irradiated with 0.5 Gy or 3 Gy and were fixed 1 or 3 h after IR exposure. Cells were stained for EdU and PCNA foci formation. PCNA foci without EdU foci were counted. (F) Restoration of the replication block after RIF1 depletion. HeLa cells transfected with siCtrl or siRIF1 were irradiated with 3 Gy and were fixed 1 h after IR exposure. PCNA foci without EdU foci were counted. Data are the mean±s.d. of three independent experiments. *P* values indicated were calculated using a Chi-squared test. (G,H) DNA synthesis at DSB sites was measured by the colocalization of EdU with 53BP1. HeLa cells transfected with either siCtrl or siRIF1 were irradiated with 3 Gy and fixed 1 h after IR exposure. Cells were stained for EdU and 53BP1 foci formation. Frequency of 53BP1 foci colocalized with EdU were measured. Data in H are mean±s.d. and the *P* value indicated was calculated using a Student's *t*-test. Scale bar: 10 μm.

A foresighted study has previously demonstrated that RIF1 depletion in HeLa cells leads to a defect in the intra-S-phase checkpoint, as evidenced by observations of radioresistant DNA synthesis (RDS) (Silverman et al., 2004). However, RIF1 has also been characterized as an HRR suppressor (Chapman et al., 2013; Di Virgilio et al., 2013; Escribano-Díaz et al., 2013; Zimmermann et al., 2013). Thus, it remains elusive whether RIF1 functions in the intra-S-phase checkpoint and in HRR suppression simultaneously or independently. Because we showed an IR dose-dependency of RIF1-mediated HRR suppression (Fig. 2), we subsequently investigated the relationship between RDS and IR doses. To analyze the intra-S-phase checkpoint, HCT116 cells were synchronized in G1 phase by lovastatin treatment. Once released, the cells in early S phase were irradiated with 0.5 or 3 Gy of IR and S-phase progression was monitored. IR exposure of 3 Gy delayed the S-phase progression in control cells, whereas 0.5 Gy did not have any impact on the progression (Fig. S4A). In contrast, RIF1-depleted cells progressed without any delay, even after 3 Gy of IR (Fig. S4A). Furthermore, DNA synthesis was directly measured by 5-ethyl-2′-deoxyuridine (EdU) incorporation after IR exposure. DNA synthesis in control cells was reduced after 3 Gy but not after 0.5 Gy of IR, and this reduction was alleviated by RIF1 depletion, displaying an IR dose-dependent RDS phenotype (Fig. 4A; Fig. S4B).

The DNA synthesis rate originates from two mechanisms: replication initiation (activation of replication origins) and chain elongation (progression of the replication forks). Because elongation appears to be more radioresistant than replication initiation, as previously reported (Lavin and Schroeder, 1988), we next investigated replication initiation. To test this, we distinguished the replication forks by pulse-labeling cells with EdU followed by PCNA immunostaining (Ge and Blow, 2010). PCNA foci were categorized into two groups: (i) PCNA foci colocalized with EdU foci (origins at which replication had begun before IR exposure) and (ii) PCNA foci localized independently from EdU foci (origins at which replication was initiated after IR exposure, termed replication initiation) (Fig. 4B). The replication initiation (group ii) increased during S phase under non-irradiation conditions and, conversely, the number of origins already activated (group i) gradually decreased as DNA synthesis at activated replicons was completed (Fig. 4C,D). When cells were irradiated with 3 Gy of IR, the number of replication initiations was significantly reduced 1 h after IR exposure; however, this number was restored after 3 h (Fig. 4E). Interestingly, RIF1 depletion alleviated the suppression of replication initiation upon 3 Gy of IR, restoring it to the levels observed in non-irradiated cells (Fig. 4F), indicating that RIF1 suppresses replication initiation after IR exposure. Because Doksani et al. (2009) reported that DSBs trigger firing of a dormant-origin near the DSB site, the effects of RIF1 deficiency on this type of firing was tested. To detect the origin firing at DSB sites, we observed EdU incorporation at 53BP1 foci, which indicate DSB sites. After 3 Gy of IR, DNA synthesis rarely occurred at DSB sites in control cells, but increased more than 10-fold in RIF1-depleted cells (Fig. 4G,H). These results strongly suggest that RIF1 has a role in inhibiting replication initiation near DSB sites in cells exposed to high doses of IR. Consistent with previous work, RIF1 formed nuclear foci in S-phase cells upon 3 Gy of IR (Silverman et al., 2004) and this foci formation was inhibited by treatment with an ATM inhibitor or by NBS1 (also known as NBN) depletion (Fig. S4C–E).

### MCM helicase is a possible molecular link between replication and HRR

**Fig. 5.**
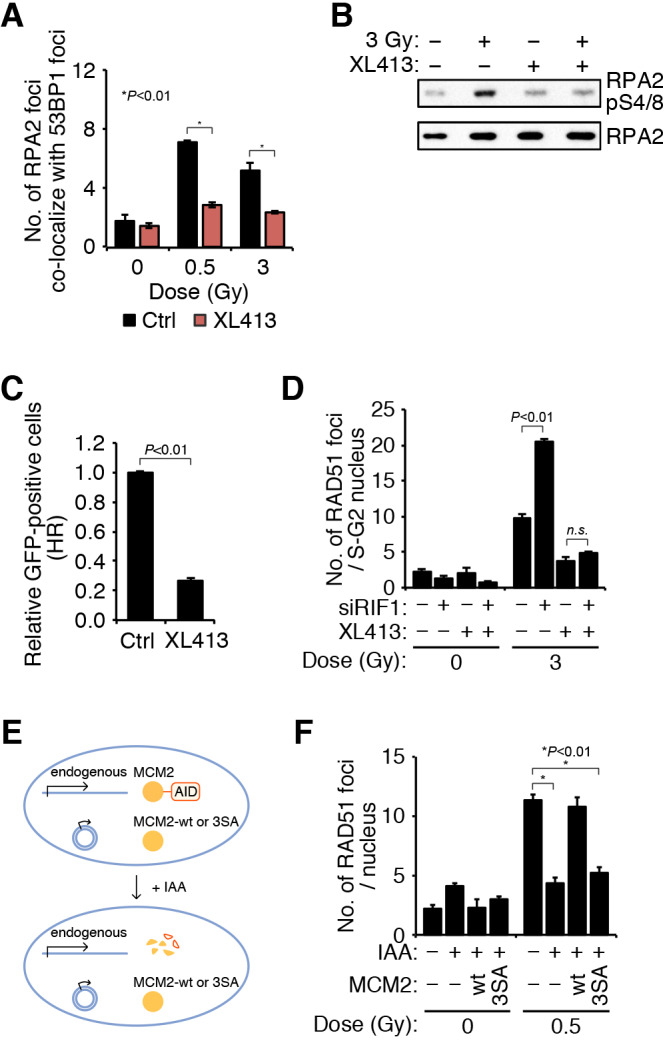
**Dephosphorylation of MCM2 inhibits DNA end resection in HRR.** (A,B) Inhibition of DNA end resection by the DDK kinase inhibitor XL413. HeLa cells were treated with XL413 for 2 h prior to irradiation and collected 1 h after IR exposure. RPA2 focus formation was observed as in Fig. 2D. RPA2pS4/S8 and RPA2 proteins were detected by western blotting as in Fig. 2E. Data shown in A are mean±s.d. (C) Decrease in functional HRR at an I-SceI-induced two-ended DSB in the presence of XL413. After transfection with pCBASce, HeLa cells were treated with XL413 for 10 h. The DR-GFP assay was performed as in Fig. 1B. Data shown are mean±s.d. (D) Inhibition of IR-induced RAD51 focus formation by XL413. HeLa cells, transfected with siCtrl or siRIF1 were treated with XL413 for 2 h prior to irradiation. RAD51 focus formation was detected using anti-RAD51 antibodies 1 h after exposure to 3 Gy of IR. Data represent the mean±s.d. of three independent experiments. (E) Construction of MCM2 rescue experiment. Endogenous MCM2 was tagged with an auxin-inducible degron (AID), which resulted in degradation upon addition of IAA allowing the activity of wild-type (wt) or phospho-dead mutant (3SA) MCM2 to be assayed. (F) IR-induced RAD51 focus formation in MCM2 depleted, wt- or 3SA mutant-expressing cells. HCT116 cells were transfected with MCM2 wild type- or MCM2-3SA mutant-expressing vectors. Endogenous MCM2 was depleted using the AID system, and RAD51 focus formation was detected using anti-RAD51 antibodies 1 h after exposure to 0.5 Gy of IR. Data represent the mean±s.d. of two independent experiments. *P* values indicated in A,D and F were calculated using a two-way ANOVA followed by Tukey's multiple comparisons test. The *P* value in C was calculated using a Student's *t*-test

Because RIF1 appeared to mediate inhibition of both replication initiation and HRR upon high-dose IR exposure, the effect of replication blockage in HRR was tested. Transient treatment of cells with a DNA polymerase inhibitor, aphidicolin, abolished RAD51 focus formation in S- and G2-phase cells, suggesting an important role of replication in HRR (Fig. S5A,B). Recent studies reported that RIF1 binds to PP1 phosphatase and inactivates MCM helicase via the dephosphorylation of MCM2 and MCM4, blocking replication initiation (Alver et al., 2017; Hiraga et al., 2017). Indeed, the phosphorylation of MCM2 was markedly enhanced by RIF1 depletion, which was accompanied by a restoration of the replication block (Fig. 4F; Fig. S5C). Immunoprecipitation of RIF1 showed that the exposure to 3 Gy of IR promoted physical interaction between RIF1 and MCM2, implying that RIF1 might dephosphorylate MCM2 in this condition (Fig. S5D,E). This interaction is consistent with the obtained results, in which HRR inhibition was marginal after 0.5 Gy, but significant after 3 Gy of IR (Fig. 2A). The importance of MCM phosphorylation in HRR was demonstrated by experiments in which the CDC7 inhibitor, XL413 abolished DNA end resection and subsequent HRR (Hughes et al., 2012) (Fig. 5A–C). Treatment with XL413 suppressed RAD51 focus formation enhanced by RIF1 depletion to the level of the intact RIF1 control, implying that the phosphorylation of MCMs is a target for RIF1-dependent HRR suppression (Fig. 5D). These results prompted us to test HRR in the phospho-dead mutant of MCM2. Endogenous MCM2 protein was depleted by using the auxin-inducible degron (AID) system (Natsume et al., 2017), and wild-type MCM2 or a phopsho-dead (S40A, S53A, S108A; referred to here as 3SA) MCM2 mutant was added back (Fig. 5E). RAD51 focus formation was abolished in MCM2-depleted cells, which is consistent with our observations of HeLa cells treated with siMCM2 (Fig. S5F). RAD51 foci formation was restored by wild-type MCM2 but not by the phospho-dead MCM2 mutant, indicating that the phosphorylation of MCM2 is critical for HRR (Fig. 5F; Fig. S5G).

Because the activity of MCM helicase is tightly regulated and only acts on chromatin during S phase (Chuang et al., 2009; Kang et al., 2012), we asked whether RIF1-dependent HRR suppression occurs only in S phase. To test this idea, HeLa cells were synchronized in G2 phase using a CDK inhibitor, RO-3306 (Vassilev et al., 2006). Cell cycle analysis with EdU and propidium iodide (PI) staining showed that almost all cells were arrested in G2 phase at 24 h after RO-3306 addition (Fig. S6A). RAD51 focus formation after treatment with 3 Gy was not rescued by RIF1 depletion in G2-phase cells, which was in contrast to the rescue observed in an asynchronous population containing S-phase cells (Fig. S6B,C). Altogether, our results suggest that RIF1 has a role in the suppression of HRR only in S phase, during which RIF1 dephosphorylates MCMs and inhibits replication initiation after a high dose of IR.

## DISCUSSION

**Fig. 6.**
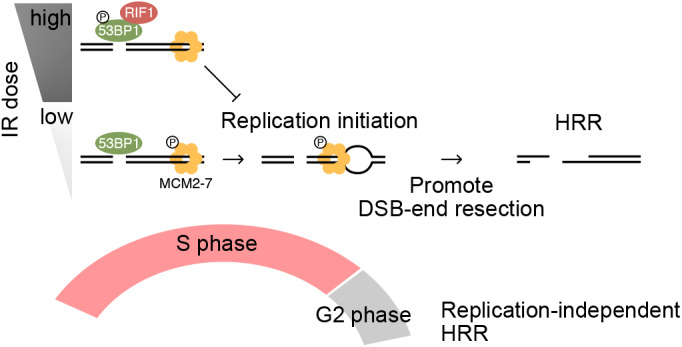
**Model of RIF1-mediated HRR suppression in S phase.** RIF1 accumulates at DSB sites and suppresses DNA replication initiation only after a high dose of IR. DNA replication promotes DNA end resection, RAD51 accumulation and HRR, whereas RIF1 inhibits both DNA replication and HRR. This regulatory mechanism is specific for S phase, in which replication is active.

This study demonstrated an IR dose-dependent suppression of HRR, in which RIF1 accumulated at DSBs and protected DNA ends from resection by suppressing replication initiation (Fig. 6). Although the protective role of RIF1 is the same as that in G1 phase (Chapman et al., 2013; Di Virgilio et al., 2013; Escribano-Díaz et al., 2013; Zimmermann et al., 2013), we found that the inhibition of HRR significantly depended on IR dose. Because of the antagonistic effect of BRCA1 activity at low dose ranges, RIF1-mediated HRR inhibition seemed to have no or little effect after exposure to a low IR dose such as 0.5 Gy (Fig. 2). RIF1 is considered a key protein in the repair choice between HRR and NHEJ, and is expected to promote NHEJ through HRR inhibition (Chapman et al., 2013; Di Virgilio et al., 2013; Escribano-Díaz et al., 2013; Zimmermann et al., 2013). However, NHEJ activity remained constant regardless of the IR dose, as measured with the pEJ reporter gene and ChIP assays (Fig. 1C,D). It appeared that RIF1 exclusively inhibits HRR during S phase, rather than regulating the choice between repair pathways for rejoining DSBs.

Recently, Ochs et al. (2016) showed that the number and total intensity of RAD51 foci steeply increases at low IR doses, plateaus at intermediate doses, and gradually declines after high doses such as 10 Gy in U2OS cells. They demonstrated that RAD51-dependent gene conversion, an error-free HRR, switches to RAD52-dependent single-strand annealing, a mutagenic HRR, due to a lack of 53BP1 after extremely high IR doses. Although they demonstrated a dose-dependent suppression of error-free HRR, it is still unclear whether the intermediate doses of IR normally used in radiation therapy can alter the usage of repair pathways. We showed that error-free HRR was suppressed even after intermediate doses of IR such as 3 Gy, and that this was controlled by RIF1.

Cells from patients with ataxia-telangiectasia (an ATM-deficiency disease) or Nijmegen breakage syndrome (an NBS1-deficiency disease) display abnormal intra-S-phase checkpoints and RDS (Tauchi et al., 2002). Because RIF1 accumulation is compromised by treatment with an ATM inhibitor or by NBS1 depletion (Fig. S4C–E), the RIF1-mediated temporary replication block might be a mechanical basis for these phenotypes. Indeed, a similar defect in the intra-S checkpoint is reported by others using RIF1-depleted cells, although it remains elusive how RIF1 regulates both the intra-S checkpoint and HRR in the presence of DSBs (Silverman et al., 2004). Our results suggest that RIF1 regulates the phosphorylation of MCM helicase to control both the intra-S checkpoint and HRR inhibition.

The phosphorylation of MCM helicase converts it into the active form, the CMG complex, which in turn physically interacts with Pol α-primase at replication origins to initiate replication (Gan et al., 2018). A recent paper showed that the 53BP1–RIF1 pathway recruits Pol α-primase to DSB sites through interaction with the CST complex, which contains proteins that are similar to RPA2, resulting in HRR regulation in G2 phase by Pol α-dependent fill-in. Failure in this fill-in causes hyper-resection of DSB ends (Mirman et al., 2018), and indeed RIF1 depletion showed hyper-resection in asynchronous cultured cells (Fig. 2D,E). However, our results, in which RIF1-mediated HRR suppression occurred in S phase (Fig. S6), demonstrated that this phenomenon was strongly associated with DNA replication and activation of MCM helicase. A simple explanation for the association of MCM and HRR is that the helicase activity of MCM promotes HRR, as RAD54 helicase is essential for HRR (Essers et al., 1997), although we cannot exclude other possibilities. More analyses of RIF1 and MCM helicase in each cell cycle phase are needed to verify the extensive role of RIF1 in HRR regulation.

IR-induced reproductive cell death has been reported to follow a cell survival curve with two phases: an initial upwardly concave curve (the radioresistant region) at low IR doses, and a subsequent linear monotonically declining slope (the radiosensitive region) at high IR doses (Hall and Giaccia, 2006). Moreover, this radioresistant region at low IR doses is specifically observed in S phase (Hall and Giaccia, 2006). In radiation therapy, therapeutic doses are divided into individual fractions delivering low radiation doses, typically 2 Gy (Joiner and Kogel, 2009). Normal human cells exposed to low doses within the radioresistant region are more likely to recover from IR-induced DNA damage, through a currently unknown mechanism. Our results show that this recovery might be attributed to HRR in S phase, which disappears with increasing IR doses. If this dose-limited radioresistance could be maximized in normal cells or minimized in tumor cells by the regulation of RIF1, radiation therapy might be more effective for controlling tumors in some tissues. Further studies on the IR dose-dependence of RIF1-mediated HRR inhibition will provide insights into the basic mechanisms involved in HRR and contribute to advances in clinical development.

## MATERIALS AND METHODS

### Cell culture and transfection

HeLa and U2OS cells were maintained in DMEM (D5796-500ML; Sigma-Aldrich) supplemented with 10% FBS. HCT116 cells were maintained in McCoy's 5A medium (SH30200.01; GE Healthcare) supplemented with 10% FBS, 2 mM L-glutamine, 100 U/ml penicillin, and 100 mg/ml streptomycin. Genetic engineering in HCT116 cells was undertaken as described previously (Natsume et al., 2016). To induce the degradation of RIF1-mAID, 0.1 µg/ml doxycycline and 100 µM indole-3-acetic acid (IAA, a natural auxin; Nacalai Tesque) were added to the culture medium. For the degradation of MCM2-mAID, 500 µM IAA was added to the culture medium. The DR-GFP (Maria Jasin, Memorial Sloan Kettering Cancer Center, New York, NY) or pEJ reporter gene were stably integrated into the genomes of HeLa and U2OS cells. siRNA sequences were as follows: siNBS1 #1, 5′-GUACGUUGUUGGAAGGAAA-3′; siNBS1 #2, 5′-GGGAAAGGGAUGAAGAAAA-3′; siNBS1 #3, 5′-GGACACAAAACCAGAGUUA-3′; siRIF1, 5′-GAAUGAGCCCCUAGGGAAA-3′; siMCM2, 5′-UCAUCGGAAUCCUUCACCA-3′; siBRCA1, siGENOME SMART pool M-003461-02 (Thermo Fisher Scientific).

### Reporter assay

A total of 5×10^6^ HeLa or U2OS cells containing the reporter genes were transfected with 30 μg of the pCBASce plasmid (gift from Maria Jasin) using a Gene Pulser (Bio-Rad) at 250 V and 500 μF. Cells were plated and cultured for 4 h before irradiation. Cells were irradiated with γ-rays and then cultured for 26 h (for a total of 30 h) before being analyzed with a FACSCalibur Flow Cytometer (BD). In the treatment with XL413 (SML1401; Sigma-Aldrich), transfected cells were plated in medium containing 10 μM XL413 and cultured for 10 h. Cells were washed and then cultured for 20 h (for a total of 30 h).

### Synchronization

Cells were synchronized as previously described (Zeng et al., 2009). Briefly, HeLa cells were cultured with 2 mM thymidine for 18 h, washed, and then released into fresh medium for 10 h. Cells were subsequently treated with 2 mM thymidine for 15 h, then washed, and placed into fresh medium. HCT116 cells were synchronized as described previously (Natsume et al., 2017).

### Immunofluorescence, immunoblotting, and antibodies

HeLa cells were synchronized on coverslips in S phase as described above and irradiated with γ-rays. Cells were fixed with 2% formaldehyde in PBS (for RAD51, RPA2) or 100% methanol (for RIF1, phospho-53BP1) at each post-irradiation period. Aphidicolin (A0781; Sigma-Aldrich) or XL413 was added to the culture medium at a concentration of 0.5 µg/ml or 10 μM, respectively, 2 h prior to irradiation. The primary antibodies used for immunofluorescence were RAD51 (B01P; Abnova; 1:1000), RPA2 (Ab-2; Calbiochem; 1:200), RIF1 (A300-569A; Bethyl Laboratories Inc.; 1:1000), 53BP1 (MAB3802, Merck Millipore or A300-273A, Bethyl Laboratories Inc.; 1:1000), phospho-53BP1 T543 (3428; Cell Signaling Technology; 1:1000), MCM2 (D7G11; Cell Signaling Technology; 1:1000) and CENPF (ab5; Abcam; 1:2000). The secondary antibodies were Alexa Fluor-488 and -546 (Molecular Probes; 1:500), and Alexa Fluor-647 (Life Technologies; 1:500). DNA was stained with 4′,6-diamidino-2-phenylindole (DAPI). Fluorescent images were collected with a BZ-9000 (KEYENCE) or DeltaVision deconvolution microscope (GE Healthcare). Data analyses were performed using Volocity software (PerkinElmer). The primary antibodies used for immunoblotting were phospho-RPA2 S4/S8 (A300-245A; Bethyl Laboratories Inc.; 1:5000), RPA2 (Ab-2; Calbiochem; 1:2000), ATM (GTX70103; GeneTex Inc.; 1:5000), phospho-53BP1 T543 (3428; Cell Signaling Technology; 1:5000), phospho-CHK2 T68 (2661; Cell Signaling Technology; 1:5000), BRCA1 (D-9; Santa Cruz; 1:2000), β-actin (A5316; Sigma-Aldrich; 1:20,000), phospho-MCM2 S53 (A300-756A; Bethyl Laboratories Inc.; 1:5000), MCM2 (D7G11; Cell Signaling Technology; 1:10,000) and γ-tubulin (T9026; Sigma-Aldrich; 1:10,000).

### ChIP assay

ChIP analyses were performed as previously described (Murr et al., 2005; Rodrigue et al., 2006), with minor modifications. Briefly, 6×10^6^ U2OS-DR-GFP cells were transfected with 50 μg of the pCBASce plasmid and cultured for 6 h. Cells were irradiated with 10 Gy of γ-radiation and cultured for an additional 4 h. Cells were fixed with 1% formaldehyde for 10 min and then incubated with 0.125 M glycine for 5 min. Cells were left for 10 min in solution I (10 mM HEPES pH 7.4, 10 mM EDTA, 0.5 mM EGTA and 0.75% Triton X-100). Cells were precipitated and left for 5 min in solution II (10 mM HEPES pH 7.4, 200 mM NaCl, 1 mM EDTA and 0.5 mM EGTA). The cell pellet was resuspended in Nuclei Lysis Buffer (50 mM Tris-HCl pH 8.0, 10 mM EDTA and 0.5% SDS) and sonicated to shear genomic DNA at an average length of ∼1000 bp. The lysate was centrifuged to remove debris. The supernatant was diluted at a proportion of 1:5 with IP dilution buffer (20 mM Tris-HCl pH 8.0, 150 mM NaCl, 2 mM EDTA and 0.5% Triton X-100) and pre-cleared with Protein A for 3 h. Beads were centrifuged and the supernatant was incubated overnight with 1.5 μg of anti-Ku70 (Abcam, N3H10) or anti-RAD51 (Abnova, B01P) antibodies, followed by immunoprecipitation with 30 μl of Protein A sepharose for 2 h. Beads were washed twice with 0.5 M NaCl in RIPA buffer (20 mM Tris-HCl pH 8.0, 0.5 M NaCl, 2 mM EDTA, 0.1% SDS, 0.1% sodium deoxycholate and 1% NP-40), LiCl buffer (10 mM Tris-HCl pH 8.0, 250 mM LiCl, 1 mM EDTA, 0.5% NP-40, 0.5% sodium deoxycholate) and twice with TE buffer (10 mM Tris-HCl pH 8.0, 1 mM EDTA). Beads were treated with RNase A and proteinase K, and were subsequently incubated in elution buffer (10 mM Tris-HCl pH 8.0, 300 mM NaCl, 5 mM EDTA and 0.5% SDS) at 65°C overnight. DNA was purified with the QIAquick Purification Kit (Qiagen). The amount of precipitated DNA was quantified with real-time PCR using SYBR Premix Ex Taq II (Tli RNaseH Plus) (TaKaRa Bio Inc.). The primer sequences were: 78-239F, 5′-AGAAGCCCAGGAGCAGGAG-3′; 78-239R, 5′-CCTCGCCCTTGCTCACCATG-3′; 207-415F, 5′-AAGGACGACGGCAACTACAAGAC-3′; 207-415R, 5′-TTGTGGCGGATCTTGAAGTTCACC-3′; 362-550F, 5′-CATGGCCGACAAGCAGAAGAAC-3′; 362-550R, 5′-CGCTTCTCGTTGGGGTCTTTG-3′.

### Replication foci analysis

Replication foci analysis was performed as previously described (Ge and Blow, 2010), with minor modifications. HeLa cells were synchronized in S phase as described above. Four hours after the release, cells were cultured for 10 min with 10 μM EdU to label replication foci. After washing with DMEM medium, the cells were immediately exposed to 0.5 or 3 Gy of γ-rays. Cells were permeabilized with HLS buffer (10 mM Tris-HCl pH 8.0, 0.5% NP-40 and 2.5 mM MgCl_2_) at 1 or 3 h after irradiation and fixed in methanol. Cells were stained with Alexa Fluor-488 azide, according to the manufacturer's instructions (Click-iT EdU Imaging kits, Invitrogen). Endogenous PCNA was stained with anti-PCNA antibodies (PC10; Santa Cruz, PC10, 1:200) and Alexa Fluor-546. A pulse SIM fluorescence microscope, BZ-X700 (KEYENCE) was used to visualize replication foci in S-phase cells. Images were analyzed using BZ-II Analyzer software (KEYENCE). More than 50 cells from three sets of experiments were analyzed to calculate the mean focus number.

### Chromatin fractionation

Chromatin samples were prepared as described previously (Chiu et al., 2014). Briefly, HeLa cells were suspended with 0.1% NP-40 in cytoskeleton (CSK) buffer (20 mM HEPES pH 7.4, 50 mM NaCl, 3 mM MgCl_2_ and 300 mM sucrose) and incubated on ice for 10 min. After low-speed centrifugation at 4000 rpm (1500 ***g***), the pellet was rinsed with 0.1% NP-40 in CSK buffer and centrifuged at 4000 rpm (1500 ***g***). The supernatant was then clarified by centrifugation at 13,000 rpm (15,700 ***g***) and collected as the S fraction. The pellet was resuspended in IP buffer (20 mM HEPES pH 7.4, 150 mM NaCl, 1 mM EDTA, 0.5% NP-40 and 10% glycerol) and left on ice for 20 min. After centrifugation at 13,000 rpm (15,700 ***g***), the supernatant was collected as the P1 fraction. The pellet was further resuspended in IP buffer, sonicated, and incubated with 200 units/ml DNase I in the presence of 5 µM MgCl_2_ and 1 µM CaCl_2_ at 25°C for 30 min. After centrifugation at 15,000 rpm (20,900 ***g***), the supernatant was used as the P2 fraction.

### Immunoprecipitation

Immunoprecipitation was performed as previously described (Ismail et al., 2015), with minor modifications. Briefly, HeLa cells were irradiated with γ-rays and cultured for 1 h. Cells were washed, left for 20 min in IP buffer (20 mM HEPES pH 7.4, 150 mM NaCl, 1 mM EDTA, 0.5% NP-40 and 10% glycerol), and sonicated. After centrifugation, the supernatant was incubated for 3 h with anti-RIF1 (A300-569A; Bethyl Laboratories Inc., A300-569A; 1 μg) antibodies followed by immunoprecipitation with Protein A sepharose for 2 h. Beads were washed 4 times with 0.3 M NaCl in IP buffer. Samples were heated at 95°C in sample buffer and used for western blot analysis.

### Statistical analysis and reproducibility

Statistical analyses of reporter assays, focus formation and ChIP assays were performed with the Student's *t*-test, two-way ANOVA followed by Tukey's multiple comparisons test, or the Mann–Whitney *U-*test. Statistical analyses of the replication foci studies were performed with the chi-squared test. All data from reporter assays and focus formation were pooled from two or three independent experiments. All western blot analyses were performed at least twice.

## Supplementary Material

Supplementary information

Reviewer comments
