# Supplementary material for: RIF1 controls replication initiation and homologous recombination repair in a radiation dose-dependent manner
Source: J Cell Sci. 2020 Jun 22;133(12):jcs240036. doi: 10.1242/jcs.240036 (PMC7328141; doi:10.1242/jcs.240036)
Supplement: Supplementary information [file joces-133-240036-s1.pdf]

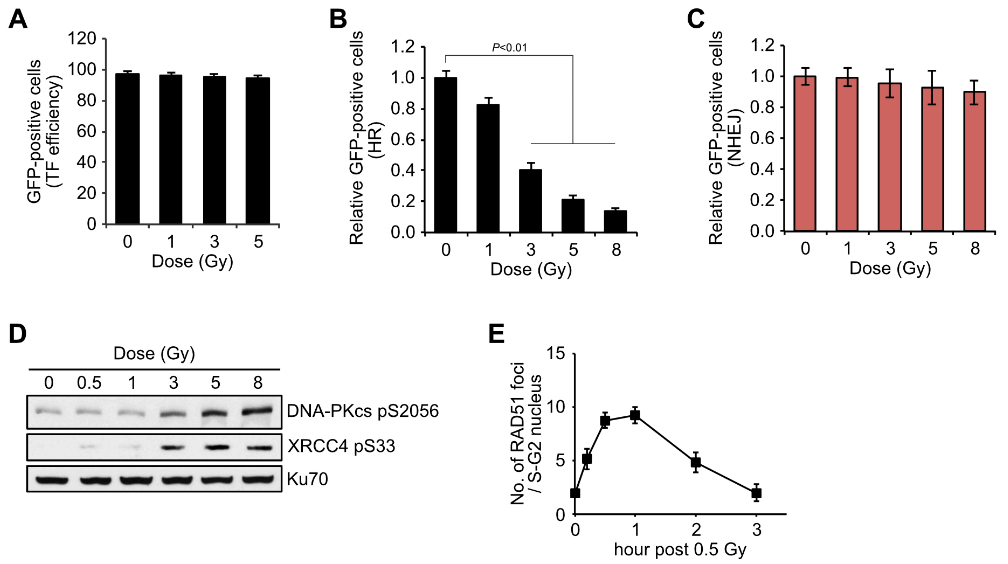

Figure S1. Activities of HRR and NHEJ after radiation. (A) No alterations in transfection efficiency after irradiation. HeLa cells transfected with pEGFP-N1 vector (Clontech) and exposed to the indicated doses of IR. The frequencies of GFP-expressing cells were measured as transfection efficiencies after irradiation. (B) Decrease in HRR after irradiation. U2OS cells harboring a DR-GFP reporter gene were irradiated and assayed for HRR activity as in Fig. 1B. Data represent the means of three independent experiments ±SDs (Student's *t* test). (C) No significant alterations in NHEJ repair. U2OS cells harboring a pEJ reporter gene were assayed for NHEJ repair as in Fig. 1C. (D) Radiation dose-dependent phosphorylation of NHEJ factors, DNA-PKcs, and XRCC4. HeLa cells irradiated with each represented dose of IR were collected 1 hour after IR and prepared for western blot with phospho-specific antibodies. Ku70 was used as a loading control. (E) Time course of RAD51 focus formation after exposure to 0.5 Gy of IR. The irradiated cells were fixed at the indicated time point after IR and were stained with anti-RAD51 antibodies. Data represent the means of two independent experiments ±SDs.

Journal of Cell Science • Supplementary information

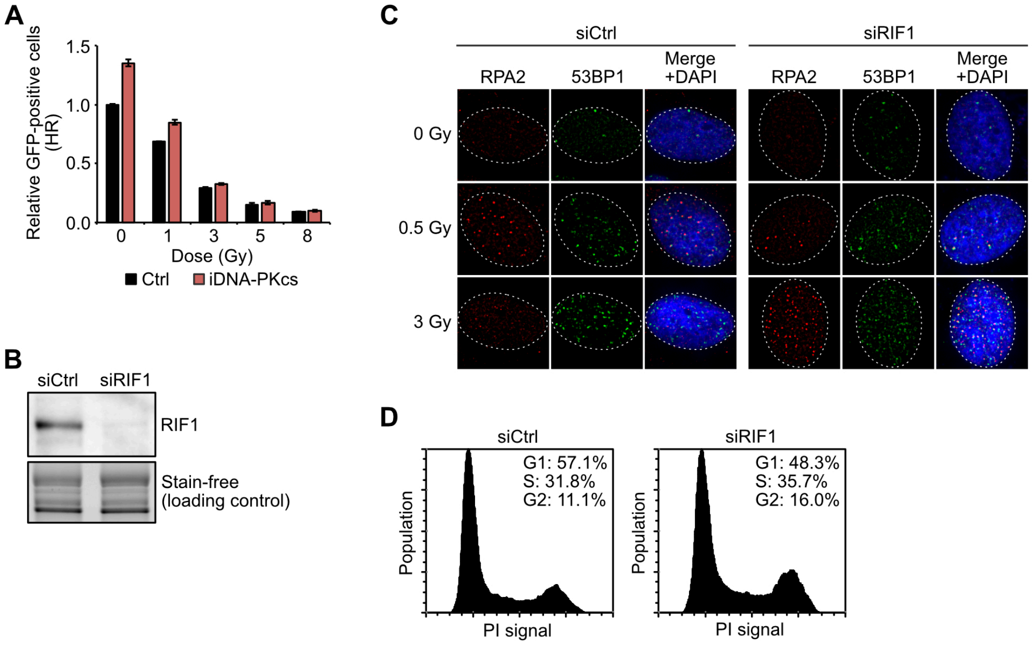

Figure S2. Rescue of HRR after high dose of radiation. (A) Inhibition of DNA-PKcs did not rescue the suppression of HRR by IR. After treatment with a DNA-PKcs specific inhibitor (NU7026 at 2 μM for 2 hours), HeLa cells were transfected with pCBASce and were irradiated with the represented dose of IR 4 hours after the transfection. The GFP+ frequency was normalized to an un-irradiated control. Data represent the means of two independent experiments ±SDs. (B) Confirmation of RIF1 depletion after transfection of siRIF1. HeLa cells were transfected with siCtrl or siRIF1 and cultured for 3 days. Expression of RIF1 was detected by western blot. (C) Refer to Fig. 2D. RPA2 focus formation at DSB sites. HeLa cells were fixed 1 hour after exposure to 0.5 or 3 Gy of IR and stained with anti-RPA2 and anti-53BP1 antibodies. 53BP1 foci were used as a marker for DSB sites. (D) Cell cycle distribution after RIF1 depletion. HeLa cells were transfected with siCtrl or siRIF1 and cultured for 3 days. After fixation, the DNA content was measured using a flow cytometer.

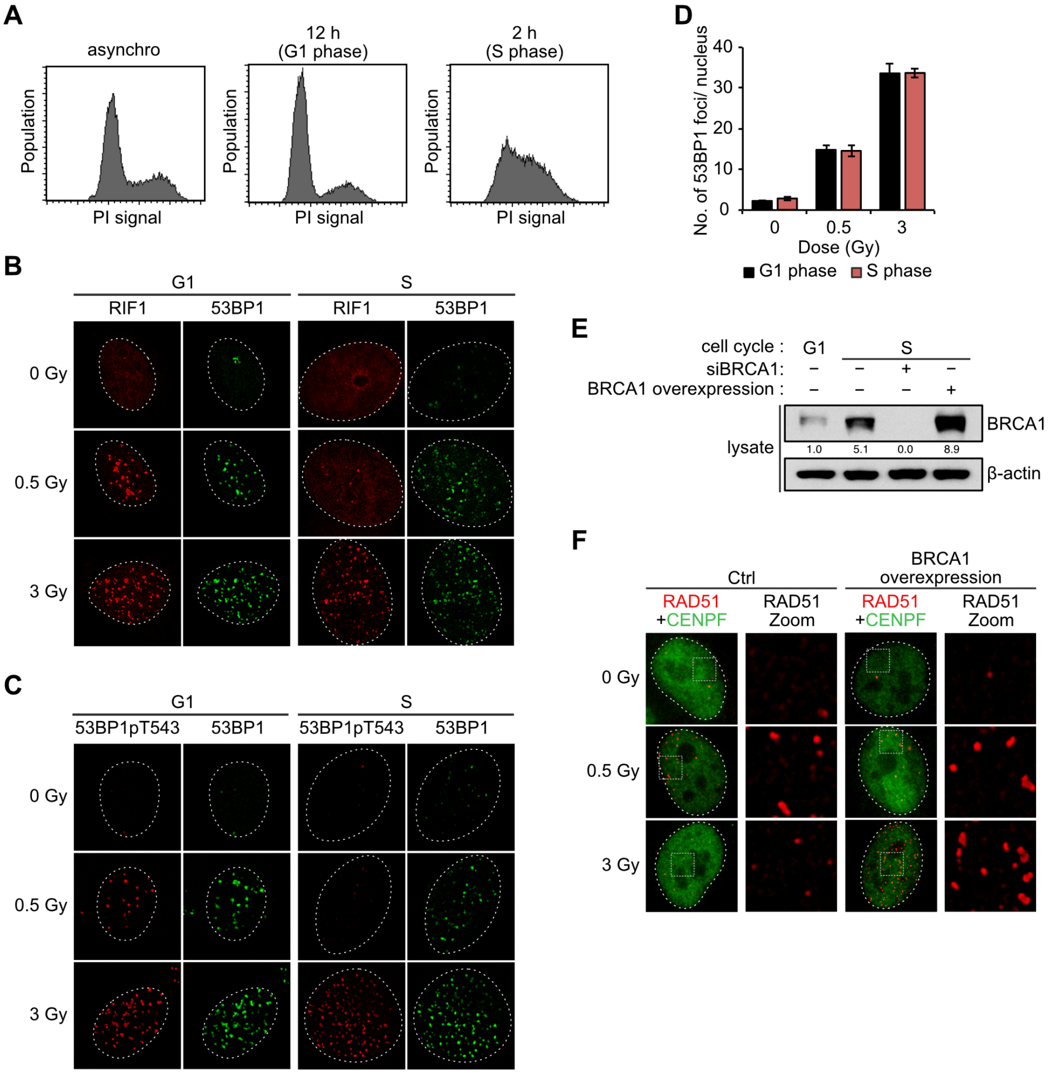

Figure S3. Cell cycle dependence of 53BP1-BRCA1 circuit. (A) Cell cycle distribution after synchronization. Cells were synchronized with a double thymidine block and collected 12 hours (for G1 phase) or 2 hours (for S phase) after release. After fixation, the DNA content was measured using a flow cytometer. (B, C) Focus formation of phosho-53BP1, RIF1, and 53BP1 in G1 or S phase cells. HeLa cells were synchronized in G1 or S phases and irradiated with 0.5 or 3 Gy of IR. Cells were stained with antibodies against RIF1, phospho-53BP1 and 53BP1 0.5 hour after IR. (D) Quantitative analyses of the focus formations in B, C. Data represent the means of three independent experiments ±SDs (Student's *t* test). (E) BRCA1 expression in G1 or S phase cells. HeLa cells transfected with siBRCA1 or with a BRCA1 expression vector were synchronized in G1 or S phases and BRCA1 expression levels were measured with western blot using anti-BRCA1 antibodies. The intensity of the BRCA1 band was normalized to that of a band from G1 phase. (F) Enhanced RAD51 focus formation after IR exposure of 3 Gy by

Journal of Cell Science • Supplementary information

BRCA1 overexpression. HeLa cells transfected with empty or BRCA1-expressing vectors were stained with anti-RAD51 antibodies 1 hour after an IR exposure of 0.5 or 3.0 Gy.

Journal of Cell Science • Supplementary information

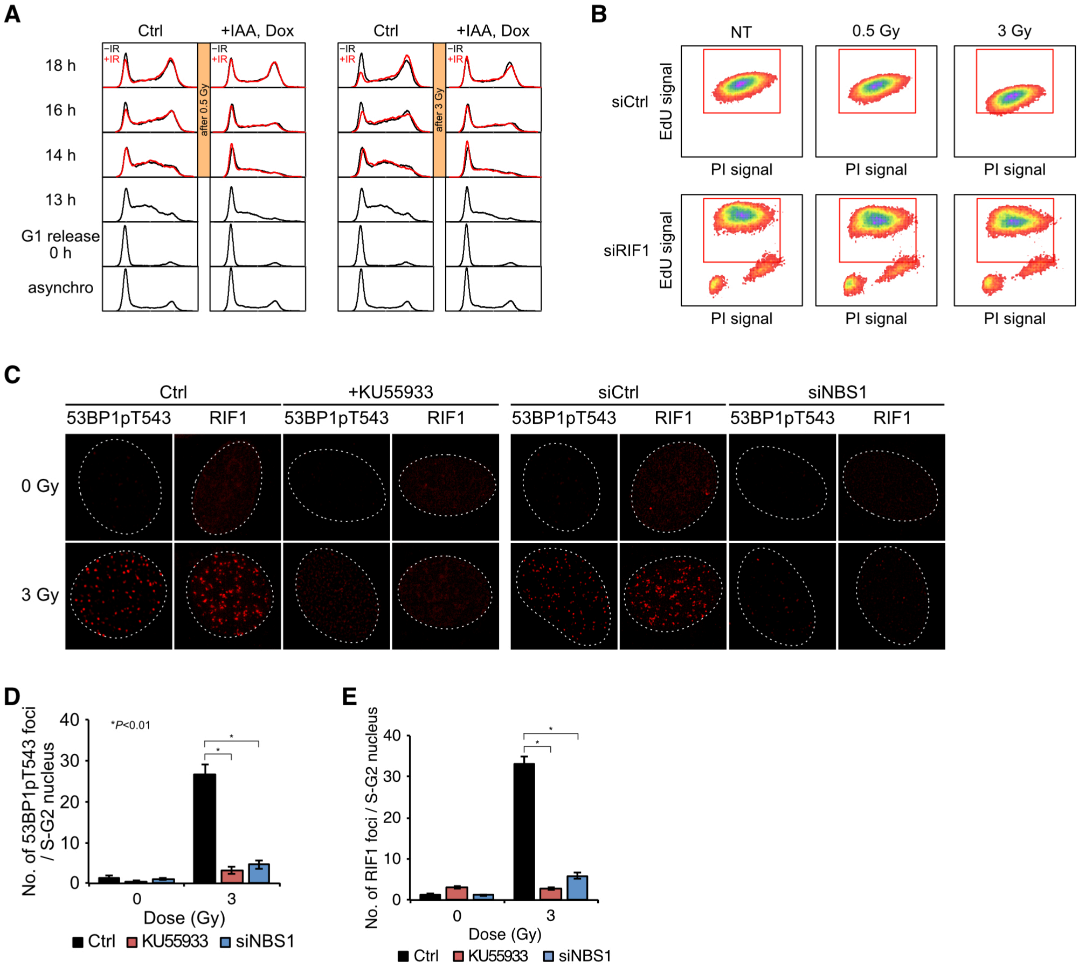

Figure S4. DNA synthesis after radiation is regulated by ATM-NBS1-RIF1 axis. (A) HCT116-RIF1mAID cells were synchronized with lovastatin and released into G1 phase. Depletion of RIF1 was performed during cell cycle synchronization by treating cells with IAA and doxycycline. When a large part of the cells was in mid-S phase, cells were irradiated either with 0.5 Gy or 3 Gy of IR and collected at each represented time point. After fixation, DNA content was measured using a flow cytometer. (B) Refer to Fig. 4A. Incorporation of EdU was measured 1 hour after irradiation using a flow cytometer. (C, D, E) ATM/NBS1-dependency of phospho-53BP1 and RIF1 focus formations. HeLa cells were irradiated in the presence of 10 μM of an ATM inhibitor (KU55933) or after transfection with siNBS1. Cells were fixed and stained with anti-phospho-53BP1 or anti-RIF1 antibodies 0.5 hour after exposure to 3 Gy of IR. S/G2 phase cells were determined by the expression of CENPF. Data represent the means of three independent experiments ±SDs (Student's *t* test).

Journal of Cell Science • Supplementary information

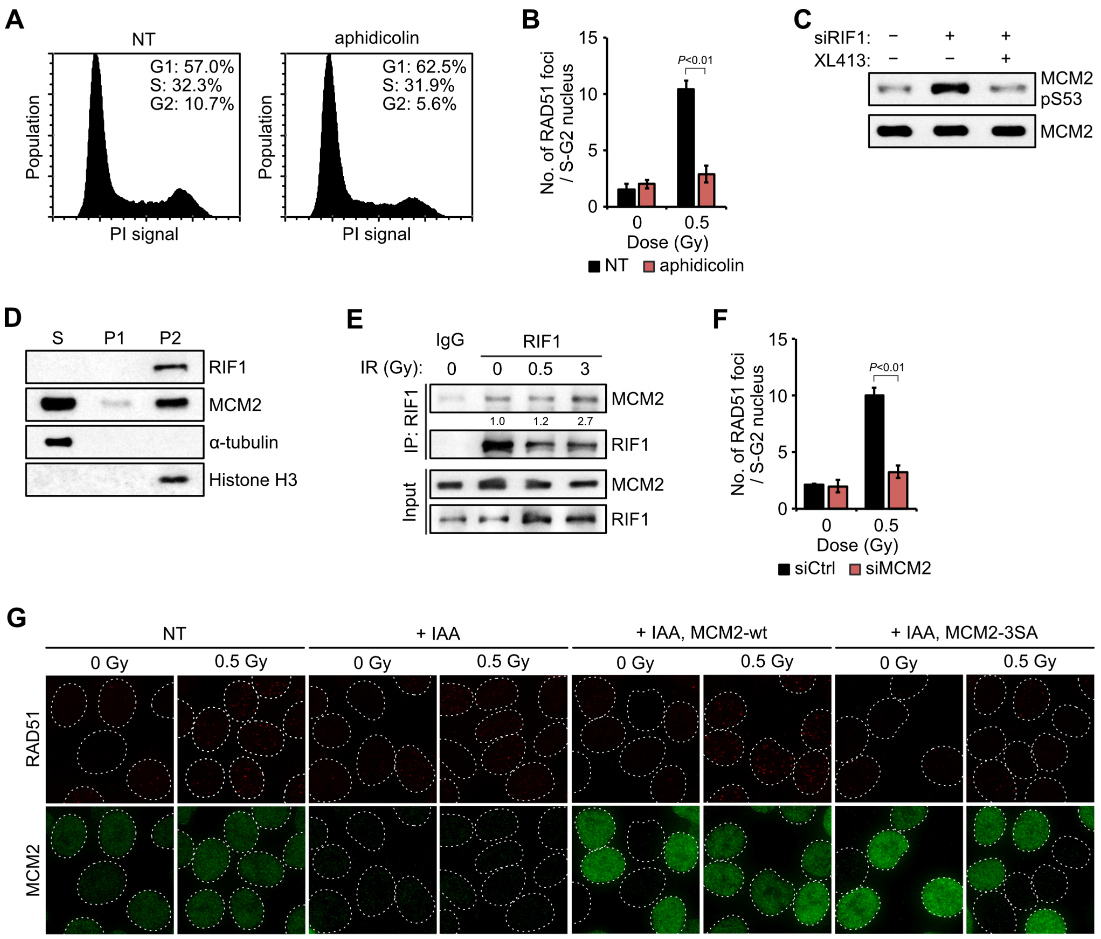

Figure S5. RIF1 targets MCM helicase to control HRR. (A) Cell cycle distribution after aphidicolin treatment for 2 hours. After fixation, the DNA content was measured using a flow cytometer. (B) Inhibition of IR-induced RAD51 focus formation by aphidicolin. HeLa cells were treated with aphidicolin for 2 hours prior to irradiation. RAD51 focus formation was detected using anti-RAD51 antibodies 1 hour after exposure to 0.5 Gy of IR. CENPF was used as a marker of the S/G2 cell cycle phases. Data represent the means of two independent experiments ±SDs. (C) Phosphorylation of MCM2 at Ser53 in HeLa cells treated with siRIF1 or with the Cdc7 inhibitor (XL413). MCM2 antibody, which recognizes MCM2 regardless of its phosphorylation status, was used as a loading control. (D) Distribution of RIF1 and MCM2 proteins. HeLa cells were fractionated into S, P1 and P2 (see Materials and Methods). Protein samples were subjected to western blot to determine RIF1 and MCM2 presences in each fraction. α-tubulin and Histone H3 were used as cytosol and chromatin markers, respectively. (E) Physical interaction between RIF1 and MCM2 in the absence or presence of DSBs. RIF1 was immunoprecipitated with anti-RIF1 antibodies from nuclear extracts of HeLa cells, which were prepared 1 hour after IR exposure. The interaction between RIF1 and MCM2 was detected

Journal of Cell Science • Supplementary information

by co-immunoprecipitation of MCM2. The numerals under each band represented the intensities of MCM2 normalized to an untreated control. (F) Inhibition of IR-induced RAD51 focus formation by MCM2 depletion. HeLa cells were transfected with siMCM2. RAD51 focus formation was detected using anti-RAD51 antibodies 1 hour after exposure to 0.5 Gy of IR. Data represent the means of two independent experiments ±SDs. (G) RAD51 foci formation in MCM2-depleted, wt- or 3SA-expressing cells. Endogenous MCM2 was depleted by AID system and wt- or 3SA-MCM2 was added back by transient transfection. RAD51 focus formation was detected using anti-RAD51 antibodies 1 hour after exposure to 0.5 Gy of IR. Two-way ANOVA, followed by Tukey's multiple comparisons test was used for (B) and (F).

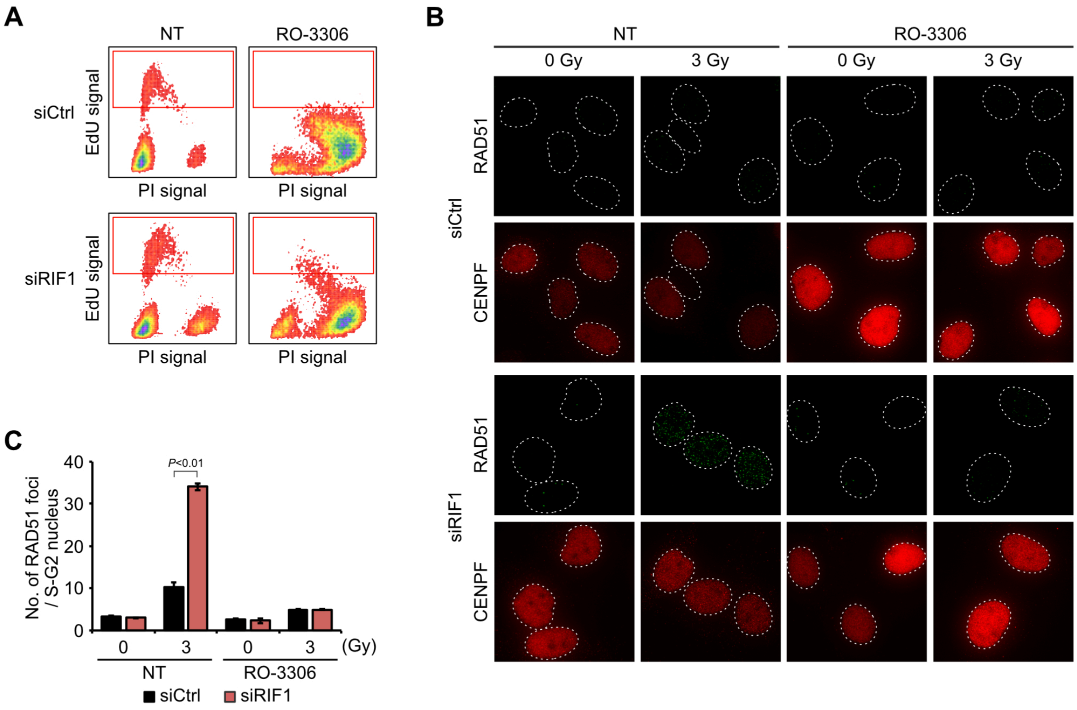

Figure S6. RAD51 foci formation in G2 phase of cell cycle. (A) HeLa cells transfected with siCtrl or siRIF1 were cultured in the presence of a CDK1 inhibitor, RO-3306 for 24 hours. Incorporation of EdU was measured using a flow cytometer. (B, C) Restoration of 3 Gy-induced RAD51 foci by RIF1 depletion was not observed in G2 phase cells. HeLa cells transfected with siCtrl or siRIF1 were synchronized with RO-3306 for 24 hours. RAD51 focus formation was detected using anti-RAD51 antibodies 1 hour after exposure to 3 Gy of IR. Data represent the means of two independent experiments ±SDs. Two-way ANOVA, followed by Tukey's multiple comparisons test was used.

Journal of Cell Science • Supplementary information
