# Supplementary material for: RIF1 controls replication initiation and homologous recombination repair in a radiation dose-dependent manner
Source: J Cell Sci. 2020 Jun 22;133(12):jcs240036. doi: 10.1242/jcs.240036 (PMC7328141; doi:10.1242/jcs.240036)
Supplement: Reviewer comments [file joces-133-240036_review_history.pdf]

Journal of Cell Science

RIF1 controls replication initiation and homologous recombination repair in a radiation dose-dependent manner

Yuichiro Saito, Junya Kobayashi, Masato T. Kanemaki and Kenshi Komatsu
DOI: 10.1242/jcs.240036

Editor: David Glover

Review timeline
Original submission: 1 October 2019
Editorial decision: 23 December 2019
First revision received: 21 March 2020
Accepted: 29 April 2020

Original submission

First decision letter

MS ID#: JOCES/2019/240036

MS TITLE: RIF1 controls replication initiation and homologous recombination repair in a radiation dose-dependent manner

AUTHORS: Yuichiro Saito, Junya Kobayashi, and Kenshi Komatsu
ARTICLE TYPE: Research Article

We have now reached a decision on the above manuscript.

To see the reviewers' reports and a copy of this decision letter, please go to: https://submit-jcs.biologists.org and click on the 'Manuscripts with Decisions' queue in the Author Area. (Corresponding author only has access to reviews.)

As you will see, the reviewers raise a number of substantial criticisms that prevent me from accepting the paper at this stage. They suggest, however, that a revised version might prove acceptable, if you can address their concerns. If you think that you can deal satisfactorily with the criticisms on revision, I would be pleased to see a revised manuscript. We would then return it to the reviewers.

Please ensure that you clearly highlight all changes made in the revised manuscript. Please avoid using 'Tracked changes' in Word files as these are lost in PDF conversion.

I should be grateful if you would also provide a point-by-point response detailing how you have dealt with the points raised by the reviewers in the 'Response to Reviewers' box. Please attend to all of the reviewers' comments. If you do not agree with any of their criticisms or suggestions please explain clearly why this is so.

Reviewer 1

Advance summary and potential significance to field

This manuscript describes a series of nice sets of experiments showing the role of roles of Rif1 in suppression of homologous-recombination repair (HRR) during S phase. Notably, this inhibition

© 2020. Published by The Company of Biologists under the terms of the Creative Commons Attribution License (https://creativecommons.org/licenses/by/4.0/).

occurs in a manner dependent on the level of the IR dose, and may involve suppression of DNA replication. The conclusions drawn by the authors are largely consistent with the data presented. The data provide novel information on Rif1-mediated regulation of HRR during S phase potentially through regulating replication initiation. The manuscript also indicates a possibility that IR dose may affect the regulation of the repair pathway choice, and adds new dimension in considering the DSB response mechanisms as well as in devising effective radiation therapy.

Comments for the author

I have concerns regarding the role of DNA replication after IR. Correlation between DNA replication (or Mcm phosphorylation/Cdc7 activity) and IR-induced HRR is likely, it is still not very clear what kind of DNA replication is occurring after 3Gy IR. The assay (PCNA foci that do not colocalize with EdU) does not tell us where the replication is occurring, and if that is authentic initiation or damage-induced replication.
In this regards, authors should consider previous publication by Marco Foiani in 2009 (Doksani et al."Replicon Dynamics, Dormant Origin Firing and Terminal Fork Integrity after Double-Strand Break Formation" Cell 137: 247-258 [2009]). In this paper, it was reported that DBS induced firing of dormant replication origins near the break site. Thus, it would be interesting to investigate whether any new initiation is induced near the break site in DR-GFP assay after DSB induction, and this is suppressed by Rif1. Since the I-SceI insertion site is known, the BrdU incorporation near the break site can be examined by BrdU-ChIP with or without Rif1 (as in Figure 1D).

Authors state that "Treatment with XL413 suppressed RAD51 focus formation enhanced by Rif1 depletion to the level of the intact Rif1 control, suggesting that the phosphorylation of MCMs is a target for Rif1-dependent HRR suppression (Fig. 5E)." However, this is still correlation and does not prove that Rif1 inhibits HRR by suppressing Cdc7-mediated phosphorylation of MCM, unless a phosphomimic form of MCM can restore the HRR suppressed by Cdc7 inhibition. Cdc7 is a promiscuous kinase in terms of its substrate, and there could be other Cdc7 target(s) that may stimulate IR-induced HRR.

Mcm2 depletion and Cdc7 inhibition reduce HRR, suggesting that DNA replication facilitates HRR. Does depletion of other replication factors including Cdc45, DNA polymerases and others cause similar effects?

Other comments:
It is interesting that Rif1 inhibits HRR in a IR-dose dependent manner. I would like some more discussion on any previous reports on potential effect of IR dose on repair pathway choice. Could there be any difference on the effect of IR dose in different cell lines?

When discussing the hyperphosphorylation of Mcm in Rif1-depleted cells,Yamazaki S. et al. EMBO J. 31: 3667-77 (2012) should be cited.

The extent of Rif1 depletion by Rif1 siRNA can be shown by western blotting somewhere.

Why is HR only partially restored by Rif1 knockdown in Figure 2A at 3Gy, while the intensity and numbers of Rad51 foci as well as the number of RPA2 foci are enhanced at the same IR dose.

It is stated in page 7 that "Immunoprecipitation of RIF1 showed that the exposure to 3 Gy of IR promoted physical interaction between RIF1 and MCM2, implying that RIF1 might dephosphorylate MCM2 in this condition (Fig. 5A, S5B, and S5C). This interaction is consistent with the obtained results, in which HRR inhibition was marginal after 0.5 Gy, but significant after 3 Gy of IR (Fig. 2A)." I would like to see if Mcm2 phosphorylation is indeed reduced after IR by its association with Rif1 (blot with Mcm2 pS53).

<u>Reviewer 2</u>

Advance summary and potential significance to field

In this manuscript, the authors described a dose-dependent inhibitory effect of Ionizing Radiation (IR) of Homologous recombination that is mediated by RIF1. The main results in the paper show

© 2020. Published by The Company of Biologists under the terms of the Creative Commons Attribution License (https://creativecommons.org/licenses/by/4.0/).

convincingly that at higher doses resection and recombination seems to be limited, and that such limitations disappear upon RIF1 depletion. This relates with the known role of RIF1 antagonizing resection in a cell cycle-regulated manner. Indeed, whereas in G1 cells RIF1 and phospho-53BP1 readily form foci at any IR dose, in S phase cells those inhibitory foci are only observed at higher doses. Additionally, the authors show that a similar effect is observed for RIF1 control of the intra-S checkpoint, been only apparent upon higher doses of radiation. Finally, the authors claim that those two events are linked, suggesting that is indeed the control of MCM phosphorylation/dephosphorylation by RIF1 what regulates resection and recombination.

Comments for the author

Whereas the observation of a dose dependent inhibition of HR by IR that relies on RIF1 is clear, the mechanistical connection with MCM is still quite far-fetched. The authors should improve this mechanistical connection in order to publish the manuscript in JCS.
Specific points to be addressed are the following:

1) Despite the obvious involvement of cell cycle on the regulation of these processes, the authors do not show any FACs profile of cells depleted or not for RIF1. In principle, and in agreement with the literature and the authors model, many, if not all, the presented results might be explained with changes in cell cycle profiles.
2) The connection with the replication upon IR is the weakest part of the paper. One major concern is that cells depleted for RIF1 are not entering S phase as they should (Figure 4). Indeed, a big proportion of the cells simply stay arrested in G1. Double thymidine block arrest cells by triggering the DNA damage checkpoint, so I will suggest the authors to repeat those experiments with a thymidine-nocodazole block.
3) The connection with the MCM phosphorylation, based on the effect of a DDK inhibitor, is not convincing at all. Inhibition of Cdc7 will affect MCM phosphorylation, but will also alter hundreds of other events in the cell. If the authors want to make such connection, they will need to prove it with a non-phosphoritable form of the MCM. Otherwise, the data just suggest that RIF1 plays two different roles upon exposure to high doses of IR, one for recombination and one for replication. Indeed, the model does not agree with the available data. If the effect of RIF1 antagonizing resection relies on MCM, why it is relocated to DSBs upon radiation? How will replication and the MCMs affect resection? There is not an obvious link for that.
4) The model is interesting, as it will signify that at higher doses the cells have a mechanism that will favor NHEJ, likely to avoid illegitimate recombination. But there are alternative explanations. One possibility is that when there are too many breaks, there is a limitation on the abundance of resection/recombination factors, that will be spread more thinly (less molecules per break) and it is easier for RIF1 to antagonize them. NHEJ proteins are notoriously abundant, while recombination factors are scarcer. This explain why simply by overexpressing BRCA1 the effect is gone.
5) Finally, it baffles me the differences observed by the authors between S and G2 for RAD51 foci (Figure S5F). They propose that the effect of RIF1 is only asserted in S, but not G2. However, this kind of contradict what most people sees and even some of the authors data. I guess that the number of cells in S phase at a given moment will be less or at much equal than the number of cells in G2 (again, FACs are missing to know the exact ratio). Thus, I will think that the data in figure 2C mainly reflect the effect of RIF1 in G2 cells. However, in figure 2C the ratio between control and siRIF1 is 1:2.5 but in figure S5F 1:2 in S and no difference in G2. If the authors are right I will expect a much bigger effect when S phase cells are considered alone than when they are considered as part of the S-G2 pack, as G2 cells will diminish the differences. This S-phase specific effect has to be cemented further, as it goes against the established model in which RIF1 has a similar role in S and G2.

Additional points:
Some statistical analyses are missing, especially in figures with more than two categories. For example, Figure 3D at 0.5 the authors compare siCtrl and siBRCA1 and at 3 siCtrl and BRCA1 overexpression. All three conditions should be compared in both cases. Also, for those graphs with two or more categories, a two-way ANOVA test is probably more relevant than individual Student's t test.

© 2020. Published by The Company of Biologists under the terms of the Creative Commons Attribution License (https://creativecommons.org/licenses/by/4.0/).

First revision

Author response to reviewers' comments

Reviewer 1

Authors should consider previous publication by Marco Foiani in 2009 (Doksani et al."Replicon Dynamics, Dormant Origin Firing and Terminal Fork Integrity after Double-Strand Break Formation" Cell 137: 247-258 [2009]). It would be interesting to investigate whether any new initiation is induced near the break site in DR-GFP assay after DSB induction, and this is suppressed by Rif1.

I greatly appreciate your useful information. The paper (Doksani et al. Cell 137, 247-258, 2009) is cited in line 27 on page 7. We investigated whether any new initiation is induced near the IR-induced DSB sites, instead of DR-GFP. The new initiation was rarely occurred near the sites, because of IR suppression of replication. However, it increased more than 10-fold in RIF1-depleted cells (see Figs 4G and 4H), which is the same as that of DR-GFP. This result strengthened our conclusion that the origin firing at DSB sites is suppressed by IR through RIF1.

This is still correlation and does not prove that Rif1 inhibits HRR by suppressing Cdc7-mediated phosphorylation of MCM, unless a phosphomimic form of MCM can restore the HRR suppressed by Cdc7 inhibition.

I think the Cdc7 inhibitor alter hundreds of other events in the cells and so, I agree your comment that it would be just correlation but not proof. Therefore, we depleted endogenous MCM2 protein by using auxin-inducible degron system and then wild type or phospho-dead mutant of MCM2 was added back (see Figs. 5E and 5F). The result showed that phospho-dead mutant failed to form RAD51 foci even after IR exposure, although the wild-type MCM2 successfully formed the RAD51 foci. This further strengthened our results that RIF1 inhibits HRR by suppressing Cdc7-mediated phosphorylation of MCMs. It was described in line 20-27 on page 8.

Does depletion of other replication factors including Cdc45, DNA polymerases and others cause similar effects?

According to your suggestion, the effect of replication inhibition on the HRR was tested by alternative method. We used an inhibitor of the polymerase, aphidicolin, to inhibit replication, which showed the significant reduction of RAD51 focus formation similar to MCM2 depletion or addition of Cdc7 inhibitor (see Figs, S5B and S5F, Fig. 5D). This was described in line 3-7 on page 8.

I would like some more discussion on any previous reports on potential effect of IR dose on repair pathway choice. Could there be any difference on the effect of IR dose in different cell lines?

According to your suggestion, we searched any previous reports on IR-dose dependence of HRR, but we failed to find the paper other than Ochs et al. (Nat Struct Mol Biol. 714-721, 2016). Ochs's paper described that HRR gradually declined and switched to RAD52-dependent single strand annealing at a high dose, such as 10 Gy. This is different from our result, in which HRR decreased at intermediate dose, such as 3 Gy, and this decrease is observed specifically at S phase cells. The same result was also obtained by using U2OS cells (Fig. S1B), which was the same cell line as in Ochs' paper. We described the cell lines, U2OS and RPE cells, which was used in Ochs' experiment (line 22 on page 9).

When discussing the hyperphosphorylation of Mcm in Rif1-depleted cells,Yamazaki S. et al. EMBO J. 31: 3667-77 (2012) should be cited.

We missed the citation of this important paper and added it in line 23 on page 3.

© 2020. Published by The Company of Biologists under the terms of the Creative Commons Attribution License (https://creativecommons.org/licenses/by/4.0/).

The extent of Rif1 depletion by Rif1 siRNA can be shown by western blotting somewhere.

According to your indication, the western blotting was added in Fig. S2B.

Why is HR only partially restored by Rif1 knockdown in Figure 2A at 3Gy, while the intensity and numbers of Rad51 foci as well as the number of RPA2 foci are enhanced at the same IR dose.

I think that RAD51 focus formation is a good HRR indicator but does not necessary represent HRR functionality. The discrepancy between partial HRR in Fig. 2A and many RPA2/RAD51 foci in Figs. 2C and 2D is interpreted by the accumulation of these repair proteins at the hyper-resected single strand DNA (ssDNA), which is too long to accomplish normal HRR. This RIF1-derived hyper-resection was also reported by others (Z. Mirman, et al. Nature, 2018). This is the reason why we used functional DR-GFP assay in addition to RAD51 focus assay in this paper. We discussed about this paper in line 5-11 on page 10.

I would like to see if Mcm2 phosphorylation is indeed reduced after IR by its association with Rif1 (blot with Mcm2 pS53).

We tried the assay for the reduction of Mcm2 phosphorylation after IR exposure several times, but failed to detect its reduction. This is probably because abundant phosphorylated MCM2 in the cycling cells make difficult to detect local reduction of the phosphorylation. Alternatively, we prepared the phospho-dead MCM2 mutant for simulation of reduced MCM2 phosphorylation after IR exposure. As expected, this mutant showed the significant reduction of HRR, similar to that after 3 Gy of IR.

Reviewer 2

Despite the obvious involvement of cell cycle on the regulation of these processes, the authors do not show any FACs profile of cells depleted or not for RIF1. In principle, and in agreement with the literature and the authors model, many, if not all, the presented results might be explained with changes in cell cycle profiles.

According to the comment, we add the FACs profiles of cells depleted or not for RIF1 in new Fig. S2D. As you pointed out, RIF1-depletion altered the cell cycle distribution and increased S/G2 phase cells, while G2 phase cells were not involved in RIF1-mediated HRR (see below; new Figs. S6B and S6C). However, it is noted that there is only slight difference in the ratio of S phase cells between cells depleted and not depleted for RIF1, although RIF1 depletion significantly increased both formations of RAD51 foci and RPA2 foci. This result further supported our idea that effect of RIF1 depletion was not mediated through alteration of the cell cycle. This was described in line

Indeed, a big proportion of the cells simply stay arrested in G1. Double thymidine block arrest cells by triggering the DNA damage checkpoint, so I will suggest the authors to repeat those experiments with a thymidine-nocodazole block

Double thymidine block induces DNA damages, so I agree with your comment that it is better to use thymidine-nocodazole block instead of double thymidine block. We tested thymidine-nocodazole block, but it still accumulated RIF1-KD cells in G1 phase. Therefore, we used HCT116 cell line and synchronized cells in G1 phase by Lovastatin treatment, which is an inhibitor of the enzyme HMG CoA reductase and arrests cells in G1 phase (see Fig.S4A). This combination produced more clearly synchronized cells than our previous synchronization. This was described in line 1-5 on page 7.

Inhibition of Cdc7 will affect MCM phosphorylation, but will also alter hundreds of other events in the cell. If the authors want to make such connection, they will need to prove it with a non-phosphoritable form of the MCM.

According to your indication, the effect of replication inhibition on the HRR was tested by using non-phosphoritable form of MCM2. We depleted endogenous MCM2 protein by using auxin-inducible degron system and then wild type or phospho-dead mutant of MCM2 was added back

© 2020. Published by The Company of Biologists under the terms of the Creative Commons Attribution License (https://creativecommons.org/licenses/by/4.0/).

(see Figs. 5E and 5F). The result showed that phospho-dead mutant failed to form RAD51 foci even after IR exposure, although the wild-type

MCM2 successfully formed the RAD51 foci. This further strengthened our results that RIF1 inhibits HRR by suppressing Cdc7-mediated phosphorylation of MCMs. It was described in line 20-27 on page 8.

If the effect of RIF1 antagonizing resection relies on MCM, why it is relocated to DSBs upon radiation? How will replication and the MCMs affect resection? There is not an obvious link for that.

It was well known that IR suppresses the replication initiation, although the precise mechanism remained elusive. Our new experiment by using EdU accumulation at 53BP1 foci showed that the origin firing is occurred near DSB sites and it is suppressed by IR through RIF1 (new Figs.4G and 4H). Hence, MCM can be involved in this origin firing near DSB sites. Simple explanation for the association of MCM and resection is that helicase activity of MCM promotes HRR, as RAD54 helicase is essential for HRR (Essers J, et al. Cell 1997). It was described in line 25-32 on page 7.

One possibility is that when there are too many breaks, there is a limitation on the abundance of resection/recombination factors, that will be spread more thinly (less molecules per break) and it is easier for RIF1 to antagonize them.

Our idea is consistent with yours, in which we believe that a limitation factor is BRCA1 protein, but not RAD51. In the antagonizing balance of BRCA1 and RIF1, HRR is promoted in cells with much BRCA1 (at a low IR dose) and conversely, it is inhibited in cells with much RIF1 (at a high IR dose, such as 3 Gy). This was supported by the experiment using BRCA1-deficient cells or BRCA1-expressed cells.

I guess that the number of cells in S phase at a given moment will be less or at much equal than the number of cells in G2 (again, FACs are missing to know the exact ratio). Thus, I will think that the data in figure 2C mainly reflect the effect of RIF1 in G2 cells. This S-phase specific effect has to be cemented further, as it goes against the established model in which RIF1 has a similar role in S and G2.

According to the comment, we tested the effect of RIF1 depletion on HRR in G2 phase cells. The cells were synchronized at G2 phase by a CDK inhibitor, RO-3306. Almost all of RIF1-depleted cells accumulated at G2 phase 24 hrs after treatment with RO-3306 and then irradiated with 3 Gy to assay RAD51 focus formation. The restoration of RAD51 focus formation by RIF1-depletion was observed in S phase-containing asynchronous cells but not in G2 phase cells (new Figs. S6), which strengthened our result that RIF1 has a role in the suppression of HRR especially in S phase. It was described from line 30 on page 7 to line 2 on page 8.

Some statistical analyses are missing, especially in figures with more than two categories.

According to your indication, we analyzed the statistical significances of Figs. 2C, 2D, 3A, 3B, 3D, 3E, 3F, 5A, S4D, S4E, S5E and S6C by two-way ANOVA test. They are described in each figure.

<u>Second decision letter</u>

MS ID#: JOCES/2019/240036

MS TITLE: RIF1 controls replication initiation and homologous recombination repair in a radiation dose-dependent manner

© 2020. Published by The Company of Biologists under the terms of the Creative Commons Attribution License (https://creativecommons.org/licenses/by/4.0/).

AUTHORS: Yuichiro Saito, Junya Kobayashi, Masato T Kanemaki, and Kenshi Komatsu
ARTICLE TYPE: Research Article

I am happy to tell you that your manuscript has been accepted for publication in Journal of Cell Science, pending standard ethics checks.

Reviewer 1

Advance summary and potential significance to field

The revised manuscript responded to my comments in a satisfactory manner. The manuscript provides important novel information regarding the roles of Rif1 in potential correlation of both DNA replication and HRR.

Comments for the author

I recommend the publication of the revised manuscript in the current form in Journal of Cell Science.

Reviewer 2

Advance summary and potential significance to field

In this manuscript, the authors described a dose-dependent inhibitory effect of Ionizing Radiation (IR) of Homologous recombination that is mediated by RIF1. The main results in the paper show convincingly that at higher doses resection and recombination seems to be limited, and that such limitations disappear upon RIF1 depletion. This relates with the known role of RIF1 antagonizing resection in a cell cycle-regulated manner. Indeed, whereas in G1 cells RIF1 and phospho-53BP1 readily form foci at any IR dose, in S phase cells those inhibitory foci are only observed at higher doses. Additionally, the authors show that a similar effect is observed for RIF1 control of the intra-S checkpoint, been only apparent upon higher doses of radiation. Finally, the authors claim that those two events are linked, suggesting that is indeed the control of MCM phosphorylation/dephosphorylation by RIF1 what regulates resection and recombination.

Comments for the author

The authors have answered all my concerns satisfactorily, thus I am glad to support its acceptance in JOCES.

© 2020. Published by The Company of Biologists under the terms of the Creative Commons Attribution License (https://creativecommons.org/licenses/by/4.0/).
